# Webcams, Songs, and Vocabulary Learning: A Comparison of In-Person and Remote Data Collection as a Way of Moving Forward With Child-Language Research

**DOI:** 10.3389/fpsyg.2021.702819

**Published:** 2021-08-09

**Authors:** Giovanna Morini, Mackensie Blair

**Affiliations:** Department of Communication Sciences and Disorders, University of Delaware, Newark, DE, United States

**Keywords:** remote testing, word learning, eye-gaze measures, songs, toddlers, preschoolers

## Abstract

This article evaluates a testing procedure for collecting eye-gaze data with toddlers and preschoolers during a word-learning task. We provide feasibility and precision data by comparing performance in an in-person version of the study (conducted under controlled conditions in the lab), with performance in a virtual version in which participants completed the testing procedure from home. Our data support the feasibility of collecting remote eye-gaze data with young children, and present it as a viable alternative for conducting developmental language research when in-person interactions with participants cannot take place. Additionally, we use this methodological approach to examine a topic that has gained popularity in recent years—the role of music and songs on vocabulary learning. We provide evidence suggesting that while songs may help increase attention during a particular task, greater attention does not lead to greater learning. In fact, preschoolers show improved word-learning performance for items that were trained in a spoken sentence compared to items that were trained in a song. This means that while songs may be beneficial for increasing child engagement, spoken sentences may be best for supporting deep level learning of language concepts.

## Introduction

Over the last 50 years we have seen important shifts toward new testing paradigms that would help shape theories of language acquisition. While initially, the study of child language had been restricted to the examination of early speech productions (Brown, 1973; Shatz, 1978), the introduction of new testing techniques, such as the Intermodal Preferential Looking paradigm (IPLP) (Golinkoff et al., 1987) would allow researchers to explore processes associated with language acquisition, even before children can produce words. The IPLP measures the speed and/or accuracy of children's looking patterns to objects on a screen, and since eye-gaze is an overt behavioral response that is present early in life, it does not rely heavily on motor control (Golinkoff et al., 2013). The IPLP has been used for decades in labs across the world, and has contributed to our understanding of critical skills within language acquisition such as word learning (Hollich et al., 2000; Halberda, 2003; Newman et al., 2018) and word comprehension (Fernald et al., 2001; Swingley and Aslin, 2002; Houston-Price et al., 2007; Morini and Newman, 2019), in children as young as 6 months (Tincoff and Jusczyk, 1999; Bergelson and Swingley, 2012). But traditionally, this paradigm required participants to visit the lab, where children would be tested in a controlled environment (i.e., a quiet room with minimal distractions), using the same equipment across participants (i.e., the same screen, speakers, and video camera). Recently, unprecedented circumstances linked to the global pandemic have pushed researchers from across fields to explore new ways to collect data—as the majority of in-person testing has been halted. Many child-language researchers have turned to virtual methods as a way of accessing diverse participants, recruiting larger sample sizes, and continuing data collection in a way that remains pandemic-proof. However, many questions remain regarding the feasibility and sensitivity of data collected via remote testing. This is particularly true, when it comes to fine-grained measures such as eye-gaze and testing of young children who inherently have limited attention and cooperation spans.

As part of the present work, we developed a virtual version of the IPLP, and compared data collected in the lab under controlled conditions (pre-pandemic) to data collected virtually (during the pandemic) with children of the same age. This approach enabled us to examine a methodological aim, which focused on addressing some of the uncertainty surrounding the precision and feasibility of a remote approach. Part of the process of developing a virtual version of the IPLP involved deciding which type of language task to ask participants to complete. We chose to use a word learning task, in which participants were taught novel word-object pairings in two experimental conditions: in songs and in spoken sentences. This decision was motivated by the following factors: (i) in-person data collection for this task was underway in the lab, so we had available data that could be compared to that of children tested virtually, and (ii) little is known about the role that songs play on preschooler's ability to learn novel vocabulary items, which meant that we would have the opportunity to address a theoretical aim in addition to the methodological one.

In recent years music interventions and learning-through-song programs, including those that target vocabulary learning for children of various ages have increasingly gained popularity (Overland, 2017). Previous research examining the role of music on language learning has primarily focused on identifying shared learning mechanisms—for example, identifying similarities between music and language and the acquisition of skills across the two (Trehub and Trainor, 1993; Trehub, 2003; Brandt et al., 2012). However, there is limited work evaluating any direct benefits of music and song on the language acquisition process itself. This information can be particularly informative for caregivers, educators, and clinicians working with young children. Teaching words through songs is a practice that can be easily incorporated into everyday activities in a variety of settings (e.g., home, classroom) and that is, in fact, widely used. Though a popular practice, we have very little empirical data on the impact of music and song on language learning.

In fact, narrowing down a concrete definition of *what* music is and *how* its features might facilitate learning across domains has proven to be remarkably hard (Cross and Morley, 2008). Music has been described as a “universal feature of human cognition,” and it can be found universally across human cultures (Brandt et al., 2012). Music, like language, expresses rhythm, emotion, and meaning, and can help convey information in attention-grabbing ways, which might be especially useful for the learning process in young children (Simpson and Keen, 2009). There is considerable evidence suggesting that certain speech registers (e.g., infant-directed speech—IDS) are characterized by a slow speaking rate, high pitch, long vowels, greater rhythmicity and repetition (Stern et al., 1982, 1983; Fernald and Simon, 1984)—making this type of speech appear more “musical” compared to adult-directed speech (ADS) (Fernald, 1992). Furthermore, young children show a robust preference for IDS over ADS (Frank et al., 2020), and there is evidence suggesting that during the beginning stages of vocabulary learning, IDS may facilitate the acquisition and recognition of words (Thiessen et al., 2005; Singh et al., 2009; Ma et al., 2011). Similarly, certain forms of music (infant-directed versions of signing in particular) have overlapping features with IDS—including a slow tempo, high pitch, and repetition (Trainor et al., 1997; Trehub et al., 1997a,b; Trehub and Trainor, 1998). These shared characteristics would suggest that perhaps children's songs, like IDS, might facilitate vocabulary learning. Nevertheless, there has been an ongoing debate regarding differences and similarities in how young children process linguistic and musical features (Pinker, 1997; Jackendoff, 2009; Peretz, 2009).

One area that has been widely studied is the role of music and songs on attention. This is an important topic, given that attention is often described as a necessary early step in the learning process. Specifically, by relying on attention skills the learner is able to choose what information from the environment is relevant (and needs to be processed), and what information should be ignored because is not relevant to complete the task at hand (McDowd, 2007). Previous work with infants between the ages of 5 and 10 months suggests that hearing children's songs leads to greater engagement and sustains attention compared to hearing other types of auditory signals (e.g., other types of music, IDS, or ADS) (Trainor, 1996; Corbeil et al., 2016). In another study, infants (5.5–6.5 months) attended longer to videos of their mothers singing than videos of their mothers speaking, further supporting a preference for songs over speech (Nakata and Trehub, 2004). However, Corbeil et al. (2013) examined whether specific features included in songs (and speech) might guide infants' preference for the different types of auditory stimuli. They found that children did not show a particular preference for melodic features of music and song, and instead showed a preference for happier sounding stimuli. For example, infants preferred to listen to IDS over a hummed melody, as well as happy sounding infant-directed song over more neutral IDS. Furthermore, infants showed no preference between happy-sounding IDS and infant-directed song. The role of music and songs on attention has also been studied in slightly older children. Wolfe and Noguchi (2009) presented 5-year-old children with stories either in speech or in a song modality. Some children heard the story with background auditory distractors, while others did not. Auditory distractors were presented in both modalities of story presentation. When distractors were present, participants were better able to recall information about the content of the story when the story was heard in a song, compared to the spoken condition. The authors concluded that music may increase selective attention and awareness in school-aged children. Taken together, these findings suggest that there is a robust attentional preference for songs over speech that has been documented in infancy and into early childhood. While the features that are driving this effect are not fully understood, there is some evidence suggesting that certain characteristics of the auditory signal (e.g., affect) might play a bigger role guiding infant's engagement than others (e.g., melodic changes alone).

It is important to note that showing preference for a particular auditory signal, does not necessary translate to greater learning. When it comes to the role of music and song and its relation to learning in the language domain, existing findings are mixed, and they come primarily from studies with older children who were second-language (L2) learners (Salcedo, 2010; Ludke et al., 2014; Good et al., 2015; Busse et al., 2018). In one study Coyle and Gómez Gracia (2014) presented Spanish speaking 5-year-olds who were learning English as an L2 with lessons targeting specific English vocabulary words. These lessons were taught using a popular children's song “The wheels on the bus.” The song was used to teach five target words. The children received three 30-min teaching sessions using this song. The sessions were structured as follows: the teacher first explained and identified the target words using a visual of the bus, then the teacher sang the song twice emphasizing the target words and their location (all words were part of the bus). Before each lesson children were asked to identify and produce the target vocabulary learned in the song. The authors found that children were better able to identify the target words receptively after each lesson in comparison to their performance before instruction. However, there was no change in their ability to produce the target words. These findings suggested that using a song to present novel target words facilitated receptive vocabulary, but did not lead to improved learning in expressive vocabulary. Another study with school-aged children between 10 and 14 years of age in Thailand examined incidental learning of vocabulary words in English (the participant's L2) by exposing participants to popular songs in English, and testing them on specific vocabulary words found in each of the songs (Pavia et al., 2019). The results indicated that the more the children were exposed to the songs, the better they were able to recall the target words within the songs. In addition to vocabulary learning, the use of music and songs has been found to enhance the acquisition of grammar skills in an L2. For example, Legg (2009) found that music aided 12–13-year-old students in French-learning classrooms during instruction of past tense verbs. Specifically, using a song to demonstrate and practice past-tense use led to higher scores at post-test than when a song was not used as part of the lessons.

Fewer studies have explored the role of music and song on language learning in young children's native language. Thiessen and Saffran (2009) presented infants (between 6.5 and 8 months) with a sequence of numbers either in spoken sentences or in a song. After a familiarization period, infants were presented with the same sequence of numbers, or a novel sequence to test whether or not they had learned the original number pattern. Testing always occurred in speech, regardless of the modality of familiarization. Infants showed a preference for the novel string suggesting that they could differentiate it from the trained sequence, only when familiarization had occurred in song, but not when they had been trained in speech. Another study with 11-month-olds examined infants' ability to detect changes in phonetic and melodic information within songs (Lebedeva and Kuhl, 2010). When participants were familiarized with a consistent four note melody, they were able to detect a change in the sequence of notes. However, when they were familiarized with a four-syllable spoken non-sense word, they were not able to detect a change in syllable order. In a follow-up task the authors examined whether embedding the non-sense words in a consistent melody (i.e., a song) would improve infants' ability to detect the change in syllable order. They found that, in fact, there was an increase in phonetic recognition when the non-sense words were presented in the song context. Lastly, one electrophysiological study examined whether 10-month-old Dutch-learning infants could segment target words that were presented in a song or in a speech stream during familiarization, and whether one condition would lead to better recognition of those words when they were presented in continuous speech (Snijders et al., 2020). Analyses of event-related potentials (ERPs) suggested that there was no difference in segmentation abilities across the two conditions (i.e., infants segmented words during both speech and song familiarization). Furthermore, there was no evidence that children could recognize the familiarized words during test trials following either song or speech. In other words, there was no evidence of songs providing a facilitatory effect during this particular task.

Nevertheless, in the majority of the previous studies participants were not asked to learn word-object relations; instead, they were tested on their ability to recognize auditory patterns that were presented during familiarization/training (e.g., numbers, words). But in the real world, children must go beyond simply tracking auditory patterns to expand their vocabulary; they must learn relations between specific sound patterns and a concrete referent (Stager and Werker, 1997; Werker et al., 1998, 2002). To learn a word like “apple” from the utterance “look at the apple!” children must first segment the target word from the continuous stream of speech, they must then identify the referent that corresponds to the new word, next they must encode the sequence of phonemes that make up the word, and lastly store the new word-referent association so that it can be retrieved later on (Capone and McGregor, 2005; Gupta, 2005). Furthermore, these associations must be generated and stored relatively fast in order for vocabulary growth to occur at the speed that it does; that is, children's vocabulary increases rapidly and it is not the case that children spend months or even weeks learning a single word.

Taken together, previous work has supported the notion that music and songs can facilitate children's memory for verbal material, with evidence coming primarily from second language vocabulary acquisition. However, the findings are mixed and the “song advantage” appears to be specific to some tasks but not others. Furthermore, there is limited data examining the role of songs on language development in young children's native language, and specifically the role of songs when it comes to acquiring novel word-object relations. Hence, additional research is needed to (i) confirm prior findings, and (ii) extend this work to vocabulary learning tasks that more closely resemble the word-learning process that young children face when acquiring words in the real-world.

The present study examined two main topics. As a first step, we aimed to investigate the efficacy and feasibility of a virtual version of the IPLP for studying word learning in young children. Additionally, we wanted to know whether training novel words through songs would lead to better acquisition of the word-object pairs compared to when words were trained using a spoken sentence. As part of the study, children were taught two new words that corresponded to novel objects. One of the words was trained using a spoken sentence produced in IDS prosody, while the second word was trained in a song. Children were then tested on their ability to recognize each item using a modified version of the Intermodal Preferential Looking Paradigm (Golinkoff et al., 1987). The overall design was identical to the one used by Schmale et al. (2012) and Newman et al. (2018) to examine word learning in children of a similar age. Participants completed the same task either *in-person*, or *virtually*, with the goal of answering the following questions:

1A) Can preschoolers successfully engage and provide codable usable data in a virtual IPLP task completed from home?
1B) Does the modality of the testing procedure (i.e., in-lab vs. remote testing) influence the pattern of results?
2A) Does the use of song result in different patterns of novel word learning compared to the use of spoken sentences?
2B) Does age mediate word learning accuracy in the spoken or song conditions?

## Methods

### Participants

Our sample included a total of 59 typically-developing preschoolers, divided into two age groups: (i) 29–32 month-olds (*N* = 38), and (ii) 47–50 month-olds (*N* = 21). Within the 29–32 month-old group, 29 of them were White, 4 were African American, and 5 were of mixed race. Within the 47–50 month-old group, 18 of them were White, 1 was African American, 1 was Hispanic, and 1 was of mixed race. Additional descriptive information for both age groups is presented in Table 1. Based on parental report, participants were being raised in monolingual English-speaking homes, and had not been diagnosed with any disabilities. The younger age group was selected because it is one that has been previously tested using in-person versions of the IPLP during similar word-learning tasks (Schmale et al., 2011; Newman et al., 2018), and because it is an age-range in which children are rapidly expanding their lexical skills (Fenson et al., 1994). The second age group was included to see whether the virtual version of the IPLP could also be successfully used with slightly older children. The idea being that 47–50 month-olds have had more exposure to screens and electronic devices (Certain and Kahn, 2002), and hence they might find sitting in front of a computer at home less novel/engaging, which might affect remote task performance. Additionally, 4-year-olds might approach the word-learning task differently. For example, they are now singing songs themselves regularly, and might rely more heavily on features of the song (e.g., the melody) during encoding of the word-object relations, which would lead to different patterns of performance compared to the toddlers in the younger group.

**Table 1:** Demographic information.

**Age group**		**In-person**	**Virtual**
29–32	Sample size	*N* = 19	*N* = 19
	Gender	Male = 4	Male = 9
	Age	*M* = 30.47, *SD* = 1.14	*M* = 30.36, *SD* = 1.04
	Caregiver's education (in years)	*M* = 18.11, *SD* = 2.56	*M* = 16.67, *SD* = 2.14
47–50	Sample size	*N* = 6	*N* = 15
	Gender	Male = 3	Male = 6
	Age	*M* = 48.68, *SD* = 0.93	*M* = 48.72, *SD* = 1.07
	Caregiver's education (in years)	*M* = 15.83, *SD* = 2.71	*M* = 17.8, *SD* = 2.18

Half of the participants (*N* = 19) in the 29–32 month-old group completed the study *in-person* using an in-lab version of the IPLP, prior to in-person data collection being suspended due to the COVID-19 pandemic. The other half was tested with a *virtual* version of the same task, and participants were recruited until we could match the sample size of the in-person group. Most of the participants in the 47–50 month-old group completed the study in the *virtual* modality (*n* = 15), and only a small number was able to complete testing *in person* (*n* = 6). Our initial goal was to test a total of 19 participants in the older group (to match the sample size that was used for the younger groups). However, two additional participants were scheduled by lab staff for the older group during the recruitment process, and since the appointments were completed, we decided to include them in the final sample. As part of the inclusionary criteria for children completing the task in-person, families needed to be able to visit the lab to complete a 30-min testing session. To be included in the virtual testing, participants needed to have access to a computer with a webcam and a screen size of 12 inches or greater, as well as a reliable internet connection.

### Stimuli

Two pairs of novel objects (4 objects total) were used to create the visual stimuli. In the videos the objects were waved back and forth to maintain participants' attention. Pairs of objects were matched for material (i.e., all were made of wood), size, and anticipated salience. Each object was a different solid color.

**Figure 1:**
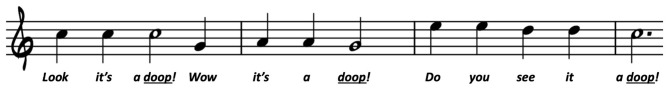
Sample of auditory stimuli heard during training trials in the song condition.

A female native speaker of American English recorded the auditory stimuli. The stimuli consisted of training sentences and test sentences. Training sentences were either *spoken* using IDS prosody or produced in a *song* to the melody of “Old Mac Donald Had A Farm” (see Figure 1). The sentences included the carrier phrase (“Look! It's a _____. Wow, it's a______. Do you see it? A _____”) followed by a target word. A total of four novel target words (to match each of the four novel objects) were presented during the study. All novel-words were one syllable long, and followed English phonotactic rules (e.g., *doop, neff* , *shoon, fim*). To ensure that the intelligibility of the context phrases was comparable across trials of the same condition, one token of each carrier phrase was selected and used for each target word. Additionally, three tokens of each target word per condition were selected (one for each of the 3-sentence carrier phrase), and cross-spliced into the sentences in the carrier phrase sequence.

Test sentences were produced by the same female speaker, and instructed children to look at one of the two objects on the screen (“Look at the _____! Do you see the _____? Where is that _____? _____!”). Note that this sequence ended with the final word presented in isolation, which was not the case for the training phrases. Additionally, all test phrases were produced in spoken sentences using IDS prosody. Once again, recordings of the different target words were cross-spliced into the same recording of the carrier phrase.

The onset of the first repetition of the target word occurred 1.4 s after the onset of the phrase; this was true for both training and testing trials. All trials were matched for amplitude and were 7.5 s in duration. Recordings were created using a Shure MV51 microphone at a 44.1 kHz sampling rate, 16-bits precision, inside a sound-attenuated booth. A sample video of the experimental task is available in a public scientific repository for this project (https://osf.io/pfazg/).

### Procedure

#### In-person

**Figure 2:**
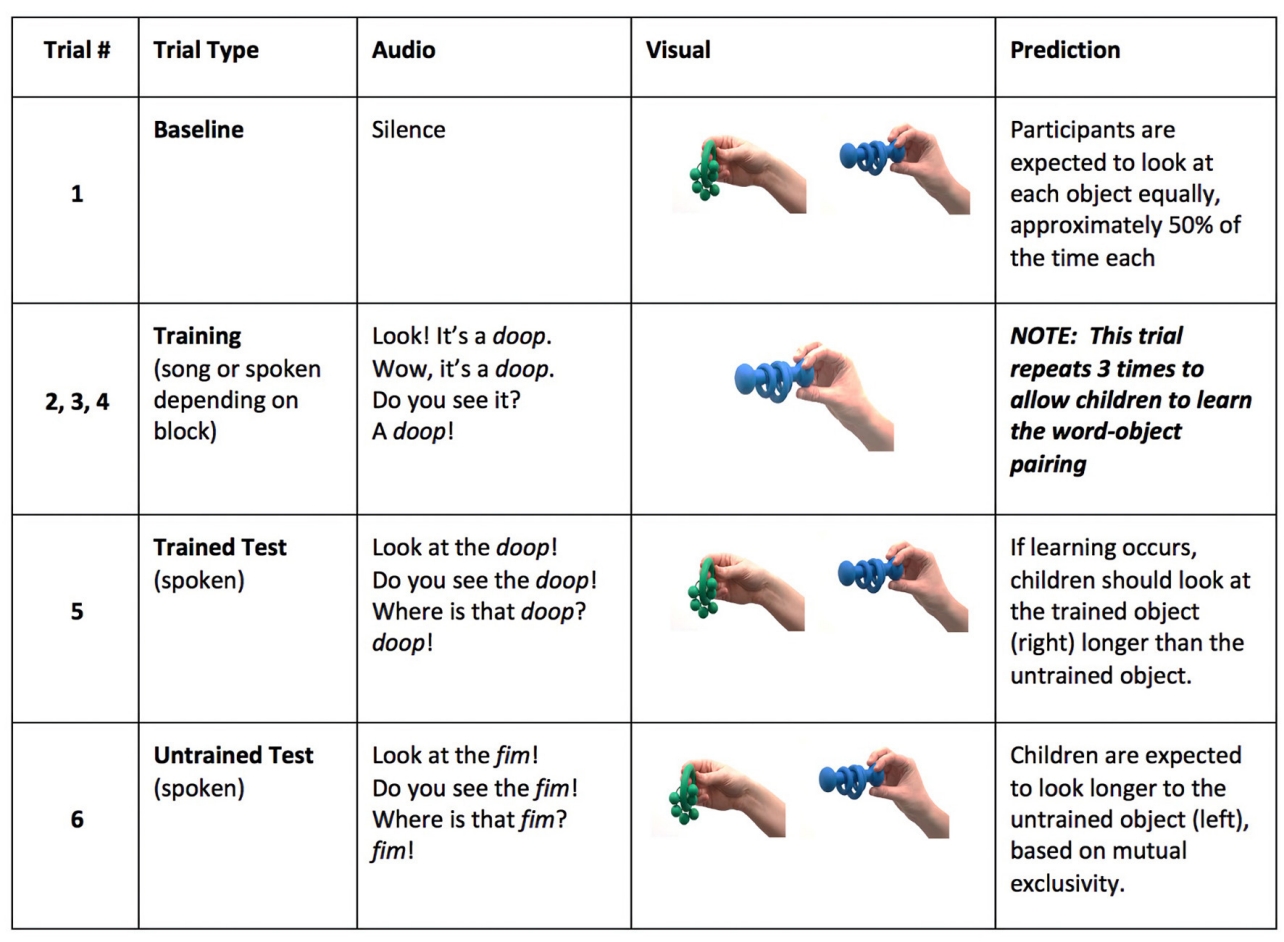
An example of the presentation of stimuli in a block.

Participants in the in-person group sat on their caregiver's lap inside a sound-attenuated booth. A 43” LCD TV screen was positioned ~5.5 feet from the participant and was used to display the videos of the novel objects on a white background. The auditory stimuli were presented through a center speaker located above the TV. Caregivers were asked either to wear headphones and listened to masking music or close their eyes during the task, to avoid biasing children's responses. An experimenter was able to see the caregiver and child with a camera throughout the duration of the study to ensure that the caregiver's headphones remained on or their eyes stayed close. The testing paradigm was divided into four testing blocks: two in the song training condition and two in the spoken training condition (see Figure 2 for an example of the presentation of stimuli in a block). Each block began with a baseline trial in which an object pair was presented on the screen without accompanying auditory stimulus. Baseline trials were included to allow us to check for object biases. After these silent trials, three training trials were then presented. During these trials a single object appeared in the center of the screen and was accompanied by sentences presented either in the song or the spoken condition. Testing for each of the word pairs occurred immediately after the training trials within each block. Blocks 1 and 2 each taught a new word: one in the song, and one in the spoken condition, and then tested that learning on the two test trials, with one trial asking for the trained object and the other asking participants to look at a novel object. Blocks 3 and 4 were an exact repetition of the first two blocks. The idea behind this design is that if children have learned the trained word-object relation, they should look longer at the trained object when it is requested. Additionally, based on the principle of mutual exclusivity (Merriman and Bowman, 1989), which assumes that objects have a single label, children should look longer at the untrained object when they are asked to look at the item that was not trained. This means that the two test trials within each block assessed successful learning of the trained word–object pairing via mutual exclusivity for the untrained test, and through direct recall of the information provided in the training trials for the trained test. This type of approach is necessary to control for trained object preferences that may arise as a result of seeing the trained object more times during the training phase. In order to be included in the final sample, participants needed to have completed (i.e., had usable data for) at least one block in each of the experimental conditions.

The following parameters were counterbalanced across participants: (i) which word was presented as the trained word, (ii) which type of test trial, trained or novel, was presented first at test, (iii) whether the song or the spoken condition appeared during the first and third blocks or the second and fourth blocks, and (iv) which object received which label. Additionally, the left vs. right position of objects on the screen was counterbalanced across blocks for each participant. An 8-s video of a dancing Elmo cartoon on a black background was included between trials to maintain children's attention. Since the trial videos had a white background and the attention-getter video had a black background, this led to changes in brightness detected by the camera that could be used to accurately identify the beginnings and ends of trials in the videos of the participants that were generated during testing. All trials had the same set duration (7.5 s) and automatically started after the Elmo attention-getter video was done playing. Visual stimuli appeared 0.4 s prior to the auditory stimulus, and the trials played uninterrupted from beginning to end. The Behavioral Infant and Toddler Testing System (BITTSy) (Newman et al., 2021) was used to control the stimulus presentation, and a video camera inside the testing booth was used to record videos of participants completing the task for later coding.

#### Virtual

Participants in the virtual group completed the study from home via a Zoom video call. Caregivers were asked to find a quiet room in the home and to try to avoid having any distractors present during the appointment (e.g., turning off the TV or music in the background). A detailed written testing protocol, which included step-by-step instructions to guide the appointment, as well as verbal scripts to explain the procedure to the families was generated and used for every testing session. This document is available in a public scientific repository (https://osf.io/pfazg/). This ensured that there was consistency across appointments, and made it possible to test families with varying levels of technical expertise. Experimenters received training on how to use Zoom and how to trouble-shoot issues that may arise during the appointments across different operating systems (e.g., Windows and Mac). A back-up experimenter (listed as “co-host” in the Zoom call) was always present, in case there were internet connectivity issues with the lead tester (i.e., this would avoid the call being dropped if one of the experimenters got disconnected).

The virtual appointment started with a light, camera, and audio check. Using the chat function in Zoom, the experimenter provided caregivers with a link to a 30-s video of a spinning wale with music playing (this video is available in the public scientific repository for this project: https://osf.io/pfazg/). The background color of the video changed from black to white every 5 s, allowing the experimenter to see if the changes in brightness (e.g., from black to white) were detectable via the webcam (as this would be used to identify beginnings and ends of trials during coding). If the contrasts were not noticeable, the experimenter asked the caregiver to adjust the lighting (e.g., close/open the curtains in the room, or turn on/off a lamp) and the video was played again. To test the audio, the video included music that was presented at the same intensity level as the auditory stimuli in the word-learning task. Caregivers were asked to adjust the volume on their computer if the sound was too loud or not loud enough, until they confirmed that they could hear the music at a comfortable listening level. Once all checks were completed, the experimenters turned off their cameras (so that they would not be visible to the child during the task), provided the link to the study video through the chat function in Zoom, and instructed caregivers to start recording the session locally on their computer using the native video recording application for their operating system (e.g., PhotoBooth for Macs and Camera app for Windows). Recording videos locally avoided lags in the video that would affect later coding. Instead of using BITTSy for stimulus presentation, the task was displayed in the form of a single video that contained all the trials and attention getters, and different versions of the video were created to preserve the counterbalancing described earlier. Caregivers were asked to set the video to full-screen, hit “play,” and close their eyes for the duration of the video. While completing the tasks, children sat on their caregivers' lap. Other than these changes, the experimental design was identical to the one described for the in-person group.

Once the experimental video had finished playing, the experimenters turned their cameras back on, and guided caregivers through steps on how to upload the video of the testing sessions that they had just generated using a secure file-transfer link. The experimenters remained on the Zoom call until the video had been successfully uploaded (this usually took 3–5 min).

### Data Coding

Participant videos for both the in-person and virtual testing sessions were coded offline on a frame-by-frame basis by two trained coders using Datavyu coding software (Datavyu, 2014). All coding files were checked for reliability across coders, and trials for which there was a discrepancy >0.5 s were re-coded by a third coder. The closest of the two coding files were used for final averaging. For participants in the 29–32 month-old age range, this happened on 4.4% of trials when the task was completed in-person, and on 15.4% of trials when the task was completed virtually. For participants in the 47–50 month-old age range, who primarily completed the task virtually, a third coder was needed on 14.5% of trials.

## Results

### Feasibility of the Virtual Version of the IPLP

As a first step, we examined how many analyzable trials were collected for children who completed the virtual version of the task, compared to children who had completed the in-person version. We focused on the data from the younger 29–32-month-old group first, given that we had a comparable number of participants who had completed the study in each modality. In order for a trial to be included in the final analyses, participants needed to have looked at one of the objects on the screen for a minimum of 500 ms. As discussed in an in-depth methodological review of the IPLP by Delle Luche et al. (2015), there is a great deal of variability across studies regarding the parameters that have been implemented for data rejection and determining trial inclusion. Many studies do not use or report a minimum looking criteria. However, previous work has established that it takes at least 233 ms for young children to program a saccade and produce looks that are linked to the processing of the stimulus (Zangl et al., 2005; Fernald et al., 2006, 2008). With this in mind, extremely short “looks” might not represent fixations that were intentional or directly linked to the child processing the auditory input that they just heard. While in some previous studies using the IPLP trial inclusion was also restricted to trials in which participants were looking at the attention-getter in the center of the screen at the trial onset, Delle Luche et al. (2015) point out that only about half of the studies rely on this practice. Furthermore, the use of this center-fixation criteria is primarily common in studies in which trial-start is triggered by an experimenter that is monitoring child behavior online, but less so in studies when trials are automatically interspaced (Swingley, 2003, 2007; Ramon-Casas et al., 2009). Given that (i) in our study the task was presented as part of a video that contained set durations for the attention-getter in between trials, and (ii) we were unable to trigger trial onsets, we did not apply this rule. As shown in Table 2, the number of analyzable trials was comparable for children in both the in-person and virtual modalities. This was true for the 29–32-month-old group as well as the 47–50 month-old group suggesting that the level of engagement with the task was similar across the two age groups that we tested. The same parameters for trial inclusion were applied to both age groups.

**Table 2:** Number of analyzable trials.

**Age group**		**In-person**	**Virtual**
29–32	Baseline trials	3.9	4
	Training trials	11.7	12
	Test trials	7.8	8
47–50	Baseline trials	4	3.5
	Training trials	12	10.4
	Test trials	8	6.9

We also looked at the attrition rate across in-person and virtual testing sessions. Data from an additional 20 participants were excluded from the in-person group due to technical problems (*n* = 1), side bias (*n* = 1), and fussiness (*n* = 18). This attrition rate is similar to what has been previously reported in other in-person IPLP studies that presented toddlers with a word-learning task (Schmale et al., 2012). Data from an additional seven participants were excluded from the virtual group due to technical problems (*n* = 3), environmental distractors (*n* = 1), not meeting the language exposure requirements (*n* = 1), and fussiness (*n* = 2). Fussiness was defined as inattention to the task and included both children who cried during the study or who refused to sit down and look at the screen. The attrition rate for 47–50 month-olds was comparable to what we observed with the toddlers. Specifically, data from an additional 12 participants were excluded due to technical problems (*n* = 6; all virtual appointments), environmental distractors (*n* = 1; virtual appointment), and fussiness (*n* = 5; 3 in-person and two virtual appointments). We had some initial concerns about being able to maintain young children's attention through a remote testing procedure, given that we expected there to be less control of the environment, and potentially greater distractors in children's homes while the task was being completed. Furthermore, we expected to lose a greater amount of data due to technical difficulties during the study (e.g., connectivity problems), and coding problems resulting from a greater variability in the quality of participant videos (due to webcams having different resolutions). To our surprise, the attrition rate was considerably lower for children tested in the virtual group compared to the in-person group. We found that participants appeared to be more comfortable in their home environment. For example, children tested in the lab more frequently wanted to get up and leave the testing booth, while children in the virtual group were more often content and remained seated in front of the screen for a longer duration. While there were some instances in which a distractor was present in the home and affected task completion for children in the virtual group (e.g., a dog barking, or a sibling talking during the exact time in which the IPLP task was being completed), this was not the norm. Additionally, in the virtual testing procedure, families did not need to travel to the lab, which meant that there was more flexibility to conduct testing sessions in a time-period that aligned better with children's schedules/routines (e.g., testing children right after they had woken up from a nap and were rested). As discussed in our limitations section later on, these parameters might be linked to the demographic characteristics of the sample (e.g., socioeconomic status), making it important to conduct further virtual work with more diverse groups of children.

### Differences in Performance Across Testing Modalities

Next, we wanted to evaluate actual performance on the word learning task and compare the data for children who were tested in-person, to that of children who completed the task from home. As a starting point, we examined children's looking time to the objects during the baseline (silent) trials. This was done to ensure there were no pre-existing biases. During these trials children in the in-person group looked at the object on the left on average 50% of the time (*SD* = 0.11) and the object on the right on average 50% of the time (*SD* = 0.11), which is what we would expect since they were not told which object to look at. Similarly, children in the virtual group looked at the object on the left on average 49% of the time (*SD* = 0.08) and the object on the right on average 51% of the time (*SD* = 0.08).

Accuracy was calculated based on the amount of time that the participants remained fixated on the appropriate image, as a proportion of the total time spent fixating on either of the two pictures, averaged over a time window of 300–5100 ms after the onset of the first repetition of the target word, across all test trials of the same condition. This window of analysis was longer than what has been previously used during word recognition tasks (Byers-Heinlein et al., 2017), and this was done given that in the present task children were asked to identify newly-acquired words—rather than highly familiar items (a more difficult task that required additional processing time). Fixating on the appropriate image in this case included the “trained object” on test trials when it was requested, and the “untrained object” on trials when the novel word was requested. This meant that each object was the “correct” item on one of the two test trials but not the other, and if children had learned the target words, they should accurately look at the correct object during both trial types. In fact, two-tailed t-tests indicated that there was no significant difference in accuracy between trained and untrained test trials for the *in-person* modality [*t*(18) = 2.04, *p* > 0.05, Cohen's *d* = 0.47], nor the *virtual* modality [*t*(18) = 0.68, *p* > 0.05, Cohen's *d* = 0.16]. Hence, for the subsequence analyses we collapsed across the two types of test trials.

**Figure 3:**
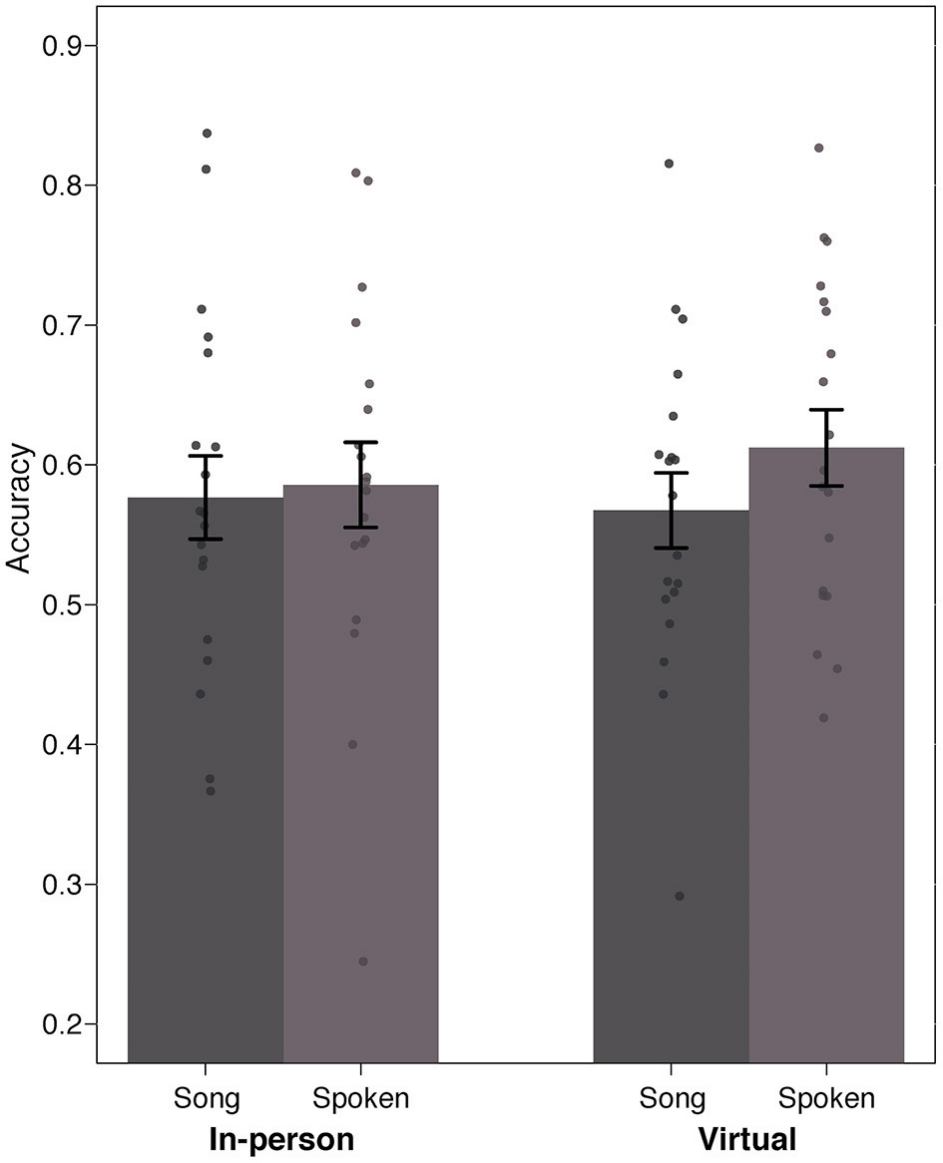
Accuracy data based on proportion of looking to the correct object across the Song and Spoken condition in 29–32 month-olds.

As shown in Figure 3, children's fixation patterns revealed that in general, accuracy was similar in the spoken condition (*in-person* modality: *M* = 0.59, *SD* = 0.13; *virtual* modality: *M* = 0.61, *SD* = 0.12) and in the song condition (*in-person* modality: *M* = 0.58, *SD* = 0.13; *virtual* modality: *M* = 0.57, *SD* = 0.11). A 2 × 2 mixed ANOVA with Modality as a between-subjects factor (in-person vs. virtual) and Training Condition as a within-subjects factor (spoken vs. song) indicated that there was no significant main effect of training condition [*F*_(1, 36)_ = 1.69, *p* > 0.05, $\eta_{p}^{2}$ = 0.048] nor modality [*F*_(1, 36)_ = 0.06, *p* > 0.05, $\eta_{p}^{2}$ = 0.001], and no significant interaction [*F*_(1, 36)_ = 0.72, *p* > 0.05, $\eta_{p}^{2}$ = 0.02]. This means that (i) the modality in which the study was completed (i.e., in the lab vs. virtually) did not affect children's performance on the task, and (ii) training words in the song condition did not lead to better performance during testing compared to when training occurred in the spoken sentences.

It is also worth noting that two-tailed single-sample *t*-tests indicated that children across the two modalities performed significantly above chance (in this case 50%) when the training occurred in the spoken condition [*in-person*: *t*(18) = 2.81, *p* < 0.05, Cohen's *d* = 0.65; *virtual*: *t*(18) = 4.11, *p* < 0.001, Cohen's *d* = 0.94], as well as in song [*in-person*: *t*(18) = 2.58, *p* < 0.05, Cohen's *d* = 0.59; *virtual*: *t*(18) = 2.51, *p* < 0.05, Cohen's *d* = 0.58].

### The Role of Song on Novel Word Learning in 29–32 Month-Olds

Another goal of the study was to evaluate whether or not using songs during training would facilitate word learning. We found no evidence of this. Our data indicated that 29–32 month-olds successfully acquired novel word-object relations during our task (as indicated by the above-chance performance), but this was equally true when training occurred in a song and in a spoken sentence. One interesting pattern, however, was that the average amount of time that children spent looking at the screen during training trials (arguably a measure of attention) was greater in the song condition than in the spoken condition. This was true for children in both the in-person modality (*song*: *M* = 6.8 s, *SD* = 0.64; *spoken*: *M* = 6.3 s, *SD* = 0.69; *t*(18) = 2.81, *p* < 0.05, two-tailed, Cohen's *d* = 0.65) as well as the virtual modality (*song*: *M* = 6.4 sec, *SD* = 0.94; *spoken*: *M* = 5.8 s, *SD* = 1.30; *t*(18) = 2.36, *p* < 0.05, two-tailed, Cohen's *d* = 0.54). While this pattern of greater “attention” when listening to songs (compared to spoken sentences) aligns with previous research on this topic (Corbeil et al., 2016), our findings would suggest that greater attention (i.e., longer looking times) during training, does not necessarily lead to better learning of the word-object mappings. To our knowledge this is the first study examining the role of song on the acquisition of word-object relations in young children's native language, and it is unclear whether the same pattern of results would extent to other age groups.

### The Role of Song on Novel Word Learning in 47–50 Month-Olds and an Examination of Age-Related Differences in Performance

To answer our last research question, we examined whether the testing procedure that we had implemented with toddlers, could also be successfully used with 47–50 month-olds to test their ability to learn novel words in the song and spoken conditions, and whether there were any age-related differences in performance between toddlers and this slightly older group. As a reminder, the majority of participants in the 47–50 month-old group completed the study virtually. Given that we found no significant difference in performance across testing modalities in our previous analyses, we collapsed across the two modalities for the subsequent results.

**Figure 4:**
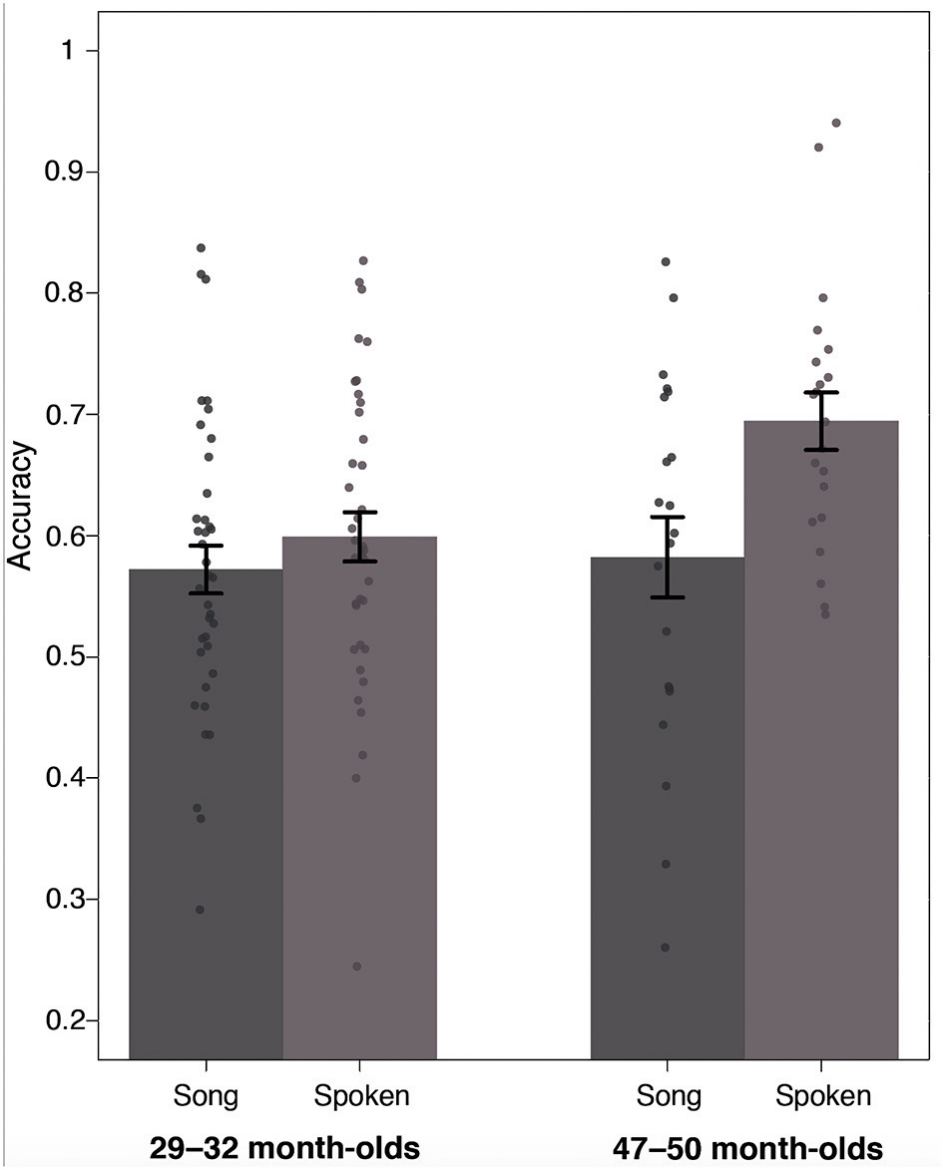
Accuracy data based on proportion of looking to the correct object across the Song and Spoken conditions for both age groups.

As an initial step, we examined looking times during baseline trials. We found that 47–50-month-olds looked at the object on the left on average 51% of the time (*SD* = 0.11) and the object on the right on average 49% of the time (*SD* = 0.11), suggesting that there were no pre-existing side biases. Accuracy during test trials was calculated using the same considerations and time window described earlier. Once again, two-tailed *t*-tests indicated that there was no significant difference in accuracy between trained and untrained test trials [*t*(20) = 2.02, *p* > 0.05, Cohen's *d* = 0.44]; therefore, we collapsed across the two trial types. As shown in Figure 4, fixation patterns revealed that surprisingly, accuracy was higher in the spoken condition (*M* = 0.69, *SD* = 0.11) than in the song condition (*M* = 0.58, *SD* = 0.15), and this difference was significant [*t*(20) = 2.71, *p* < 0.05, two-tailed, Cohen's *d* = 0.59]. Additionally, two-tailed single-sample *t*-tests indicated that accuracy for the 47–50 month-olds was significantly above chance in both the spoken [*t*(20) = 8.22, *p* < 0.001, Cohen's *d* = 1.79], and the song condition [*t*(20) = 2.48, *p* < 0.05, Cohen's *d* = 0.54], suggesting that children in this age group were also successfully learning the novel word-object pairings.

To examine possible age-related differences, we ran a 2 × 2 mixed ANOVA with Age as a between-subjects factor (29–32 vs. 47–50) and Training Condition as a within-subjects factor (spoken vs. song). This analysis indicated that there was no significant main effect of age [*F*_(1, 57)_ = 3.73, *p* > 0.05, $\eta_{p}^{2}$ = 0.04], but there was a significant main effect of condition (*F*_(1, 57)_ = 11.1*, p* < 0.001, $\eta_{p}^{2}$ = 0.07), and a significant interaction [*F*_(1, 57)_ = 4.163, *p* < 0.01, $\eta_{p}^{2}$ = 0.026]. To further explore the interaction effect, we conducted simple effects analysis. These demonstrated that when word training occurred in the song, there was no significant difference in performance between the age groups [*F*_(1, 114)_ = 0.0883, *p* > 0.05, $\eta_{p}^{2}$ = 0.001]. However, when training occurred in the spoken condition there was a significant difference between the groups [*F*_(1, 114)_ = 7.691, *p* < 0.05, $\eta_{p}^{2}$ = 0.06], with 47–50 month-old's showing higher accuracy (*M* = 0.69, *SD* = 0.11) compared to 29–32 month-olds (*M* = 0.59, *SD* = 0.12). Together, these data suggest that using a song to familiarize young children with novel words, does not lead to better learning. In fact, in our current task hearing words in the spoken sentences (during training) led to higher accuracy during testing in the case of the 47–50 month-olds. Accuracy for the song condition was still significantly above chance, which indicates that hearing words in the song did not prevent participants from acquiring the word-object relations. However, the song did not provide a “boost” in learning, as might have been expected based on the prior attention literature. We also examined whether the average amount of time that children spent looking at the screen during training trials was different for the song compared to the spoken condition (as we had seen for the 29–32 month-old group). However, this was not the case for the 47–50 month-olds [*song*: *M* = 6.2 s, *SD* = 0.92; *spoken*: *M* = 6.1 s, *SD* = 0.88; *t*(20) = 0.65, *p* > 0.05, Cohen's *d* = 0.14]; that is, although “attention” during training was the same for the two conditions, we still found greater performance during test trials in the spoken condition.

## Discussion

The present work set out to investigate the feasibility and precision of a modified version of the Intermodal Preferential Looking Paradigm, which relied on the use of virtual appointments and access to video collected through webcams in participants' homes. Previous studies using the IPLP have primarily used this measure in a controlled lab setting (Golinkoff et al., 2013); however, due to the global pandemic, many researchers have had to transition to remote testing, in order to keep developmental research activities moving forward. This sudden shift in testing practices has raised questions related to the advantages and disadvantages that come along with collecting data in more natural environments, especially when working with young children who are more easily distracted, and when dealing with fine-grained measures (such as eye-gaze). Our work contrasts data collected through a new virtual version of the IPLP, with data collected through a more established (in-person) version of this paradigm. This is a critical step for advancing developmental research and expanding testing procedures in a sustainable and reliable manner.

The methodological aim outlined above was intertwined with an additional goal to examine the role of song on young children's vocabulary learning. Previous studies examining the use of music and songs as a tool for teaching language have primarily been conducted with school-aged children in foreign language classrooms (Legg, 2009; Coyle and Gómez Gracia, 2014; Pavia et al., 2019). To our knowledge, no previous studies have directly measured whether songs can be used as a tool to facilitate vocabulary learning (specifically word-object relations) in young children who are acquiring their native language. Furthermore, it is unclear whether there might be developmental changes in how children make use of the information included in the auditory signal (e.g., features of the song), during the word learning process. Our work examined these questions with toddlers and preschoolers using a novel word learning task.

With regards to our methodological goal, data from the younger 29–32 month-old group suggest that there were no differences in performance across participants tested in person and children tested virtually. For both groups, the testing paradigm was identical. The main difference was that one group of toddlers completed the task in a controlled environment (i.e., a quiet booth in the lab)—using the same equipment across participants (i.e., the same screen, speakers, and video camera), while the other group of toddlers participated from home via a live video call—and used whatever computer screen and camera was available to them. The similarity in performance between groups supports the versatility of the IPLP as a measure that can be used in both lab and remote settings. Based on coding-reliability checks we found that a third coder was more often needed for videos collected with the virtual group, likely due to lower-resolution videos being captured through webcams compared to our in-lab camera, but this only led to an 11% increase in third-coders, which was still manageable. Furthermore, the attrition rate was actually lower for children tested in the virtual group compared to the in-person group, and we argue this was a result of (i) children being more comfortable and hence less fussy in their home environment, and (ii) the virtual testing procedure allowing us to accommodate better to children's schedules/routines since families no longer had to travel to the lab. We also tested 4-year-olds using the same task, with most participants completing the virtual version of the IPLP. Not only were children in this older group able to complete the task, but coding and attrition rates were comparable to what we had observed with the younger group. Hence, this step allowed us to extend the feasibility of the remote testing approach to slightly older children. It is worth noting that our task only took 7 min to complete, and so the brief duration likely prevented an increase in issues related to children's attention, and opportunity for distractors to interfere with testing in the home—as might have been the case had the task been longer. It is therefore important to expand this work to other tasks, to examine how different durations and dependent measures might affect the feasibility of collecting data remotely.

Our investigation also provided important insight into the role of song on the acquisition of word-object relations. Children aged 29–32-months were successful at learning novel words, but performance was the same for both words trained in the song condition, as well as in the spoken condition. In other words, we did not find evidence of a facilitatory effect during learning associated with hearing novel words in a song. Children aged 47–50-months once again were accurate in identifying novel word-object pairs that were trained during the task. However, for this older group, performance was higher for words trained in the spoken compared to the song trials. Together, these results suggest that there are age-related differences in how children make use of the auditory information they are presented with while attempting to link words with referents. They also suggest that the use of songs might not facilitate word learning in a native language for toddlers and preschoolers.

These results do not align with (i) previous findings with infants, in which songs were linked to benefits in the acquisition of auditory patterns (Thiessen and Saffran, 2009; Lebedeva and Kuhl, 2010), nor (ii) studies with school-aged children who showed a facilitatory effect of songs when learning a second language (Coyle and Gómez Gracia, 2014). There are some possible explanations for this. First, in the studies with infants, participants simply had to identify sequences of sounds. In the present study, it was necessary to make connections between the auditory patterns (in this case the novel words) and the referents during training, and subsequently rely on those relations to look at the target object on the screen during testing. Second, in the literature with children who were acquiring an L2, the songs were used across multiple training sessions over a longer period of time (i.e., there were more opportunities to hear the song), and testing was not conducted immediately after a single exposure to the training stimuli (i.e., it was more a measure of retention, rather than immediate recall of the words). This means that the tasks across studies were arguably different and were measuring different abilities. Under this view, it is important to refrain from making overarching conclusions about the role of songs across different types of learning tasks, given that benefits associated with this type of input appear to be task-specific.

There are however, some studies that have reported similar patterns to the ones observed in our data. This comes from tasks in which children were taught content knowledge information in classroom settings. Calvert and Billingsley (1998) examined preschooler's ability to learn their phone number. They found that children were more accurate at remembering their phone number when it was presented to them in speech rather than song. In that same paper, they also discussed data indicating that while repeated exposure to a song improved verbatim word-for-word memory of lyrics in an unfamiliar language (in this case incomprehensible French), it did not facilitate recall of words in a familiar language. Similar findings were identified in a study with second-grade students in which information about historical events was trained either in songs or in speech, and later assessed (Calvert, 2001). Once again, songs led to improvement in verbatim memory, but only training in the spoken condition was associated with better retention of content knowledge. The authors propose that there are different “levels of learning,” from more superficial processing of information (e.g., verbatim word-for-word memory, in which the actual meaning is not retained), to deeper learning (e.g., encoding and retrieving the details about the historical events). Furthermore, songs might be more conducive to superficial-level learning, as children may focus on superficial qualities of song (e.g., the rhyming, melody) rather than the content information.

This theoretical explanation could help us understand why preschoolers in our study had higher accuracy in the spoken condition compared to the song condition. Our task was challenging, as it required participants to understand the relation between the objects and the words to accurately look at the target object during trained test trials. In addition, children had to use that information along with their understanding of mutual exclusivity to also look at the correct object during untrained test trials. These steps likely required a deeper level of learning than if children where simply tested on their ability to recognize that they had heard the word “doop” based on verbatim memory, without knowing its meaning (i.e., what referent it corresponded to). In the case of the 29–32 month-old group, overall performance in the task was lower compared to the older participants, so it is possible that the task was simply more challenging for the younger group. In other words, given the difficulty of the task, it may not have been sensitive enough to capture differences that may exist between the use of speech and song for learning word-object relations in toddlers. We acknowledge this as a limitation of the study.

There are other elements that may have limited our findings. First, the modality of the testing trials required participants to generalize words across song and speech. As a reminder, in our paradigm children were trained in either spoken sentences or in a song (depending on the block), but all testing trials were presented in spoken sentences. This meant that in the song blocks, children had to recognize that the word “doop” that was sung during training, was the same word “doop” that was spoken during testing. We chose this methodological approach because it is one that has been used in previous studies with young children (Thiessen and Saffran, 2009). Additionally, given that in the real world children must rely on spoken sentences for oral communication and social interactions, this type of generalization is critical if songs are to be used as a way of supporting language learning. We do know, however, that infants have difficulty identifying words that they heard during familiarization when there were differences in the speech signal during testing; for example, hearing a word in a happy voice and later hearing it in a neutral or sad voice (Singh, 2008). Given that song exaggerates features of speech, there may have been a similar disadvantage at play, when children had to generalize from song to speech in our study. To examine this possibility, future work should manipulate the modality of the testing trials to see if a change that eliminates the need to generalize words in the song condition would lead to a different pattern of performance.

A second point related to the characteristics of the speech stimuli, is that sentences in the spoken condition were produced using infant-directed speech prosody. As stated in the introduction, IDS contains melodic features that make it more similar to songs compared to say adult-directed speech (ADS). The methodological decision to use IDS was made given that previous studies that used the IPLP to examine word learning in toddlers have used this type of speech register (Schmale et al., 2011; Newman et al., 2018), and because IDS has been found to increase attention and guide word learning in toddlers (Nencheva et al., 2021). Nevertheless, it is possible that adding a condition in which spoken sentences are produced in ADS might lead to even better accuracy during this type of learning task, and perhaps even lead to a difference in performance with the younger participants. This step would offer a good comparison since the spoken sentences would be less melodic and more distinct from the song condition, and would provide a better understanding of what might be driving the effects that were observed with the present data.

Third, in our study, children were only presented with a limited number of training trials, and testing was only carried out immediately after training. While this is a type of design that has been previously used in word-learning studies with children of similar ages (Schmale et al., 2012; Newman et al., 2018), it limits our ability examine whether variations in the amount of training may lead to songs providing a benefit. For example, in real-world scenarios, children have more than three exposures to a novel word-object pair. Furthermore, we only tested children on their ability to identify words immediately after being familiarized with the novel words. It is possible that additional testing that is delayed (e.g., a week after training) might provide information about the retention of information that children learned during the task, and whether songs and spoken sentences affect retention of the words differently. These questions should be explored in future work.

Fourth, the use of a familiar melody in the song condition may have posed an additional challenge. The study used the tune of “Old MacDonald had a Farm”—changing only the words of the song. Using familiar melodies and changing the lyrics to introduce new concepts is a common practice in educational settings with children of different ages (Wolfe and Hom, 1993). However, it is possible that the use of a familiar melody during training may have resulted in some level of confusion, as children could have been anticipating the familiar lyrics rather than those presented to them. Based on parent report, 100% of the children in the 47–50-month group were familiar with the song “Old MacDonald had a Farm,” as were 100% of the children in the 29–32-month virtual group. Additionally, 16 of the 19 children in the 29–32-month in-person group were familiar with the song, and parents of the remaining three children were unsure if their children knew the song. This meant that the vast majority of participants who completed the task knew the song and may have anticipated hearing the “traditional” words. While performance in the song condition was still above chance for both age groups—suggesting that the song was not preventing children from learning the word-object relations altogether—a potential boost in learning from the song may have been hampered by pre-existing expectations about the melody. An interesting follow-up study would be to use an unfamiliar melody during the training phase, as this would remove prior experience with the song lyrics.

Lastly, there are limitations associated with the demographic characteristics of the children that were included in the present work. It is important to first note that our sample included primarily children from households with mid-to-high socioeconomic status (SES). This was true for both age groups. Additionally, to participate in the virtual version of the study, families were required to have access to high-speed internet and a computer with a webcam, which limited participation opportunities for some families. Nevertheless, barriers exist for in-person studies as well. In many cases, families must have access to transportation, as well as available time during lab operating hours to visit the lab and complete the testing session. Some ways to mitigate the in-person obstacles have been to provide funds for transportation and to offer flexible testing hours. There are also potential ways of addressing barriers associated with online testing that are worth considering, which include providing families with hot spots for internet access, and offering temporary access to technological devices (e.g., loaner computers). A critical next step is therefore, to extend this work to more diverse groups of children, as it will improve our ability to generalize the results.

To conclude, findings from the present study support the feasibility of using a virtual version of the IPLP to collect remote eye-gaze data in both toddlers and preschool children. This serves as a way of continuing to move forward with developmental language research, during situations when it is not possible for in-person interactions with participants to take place. Additionally, we provide evidence suggesting that using songs during vocabulary training does not result in better learning, and that providing linguistic information to young children through spoken sentences might lead to improved outcomes. These findings hold implications not only for learning-through-song interventions, but also for instruction in educational settings. While using songs may help increase attention during a particular task, greater attention may not equate to deep-level learning. Therefore, using songs may help increase engagement (and perhaps participation), but when introducing new concepts for children to retain, using spoken sentences may be best.

## Data Availability Statement

The original contributions presented in the study are publicly available. This data can be found at: https://osf.io/qmb6t/.

## Ethics Statement

The studies involving human participants were reviewed and approved by the Institutional Review Board at the University of Delaware. Written informed consent to participate in this study was provided by the participants' legal guardian/next of kin.

## Author Contributions

Both authors listed have made a substantial, direct and intellectual contribution to the work, and approved it for publication.

## Conflict of Interest

The authors declare that the research was conducted in the absence of any commercial or financial relationships that could be construed as a potential conflict of interest.

## Publisher's Note

All claims expressed in this article are solely those of the authors and do not necessarily represent those of their affiliated organizations, or those of the publisher, the editors and the reviewers. Any product that may be evaluated in this article, or claim that may be made by its manufacturer, is not guaranteed or endorsed by the publisher.
